# Completed Genome Sequences of *Borrelia burgdorferi Sensu Stricto* B31(NRZ) and Closely Related Patient Isolates from Europe

**DOI:** 10.1128/genomeA.00637-17

**Published:** 2017-07-13

**Authors:** Gabriele Margos, Sabrina Hepner, Christoph Mang, Andreas Sing, Bernhard Liebl, Volker Fingerle

**Affiliations:** German National Reference Centre for Borrelia (NRZ), Bavarian Health and Food Safety Authority (LGL), Oberschleissheim, Germany

## Abstract

*Borrelia burgdorferi sensu stricto* is a causative agent of human Lyme borreliosis in the United States and Europe. We report here the completed genome sequences of strain B31 isolated from a tick in the United States and two closely related strains from Europe, PAli and PAbe, which were isolated from patients with erythema migrans and neuroborreliosis, respectively.

## GENOME ANNOUNCEMENT

*Borrelia burgdorferi sensu stricto* is a tick-borne pathogen that is maintained in natural transmission cycles in North America and Europe. Multilocus sequence typing (MLST) on *B. burgdorferi sensu stricto* from Europe and the United States has shown that strains recovered from field-collected ticks appear to represent different MLSTs, while there appears to be an overlap between European and North American MLSTs when strains are isolated from patients (1). Similar results were obtained when chromosomal single nucleotide polymorphisms (SNPs) were considered (2).

To understand the relationship of such strains in greater depth, whole-genome sequences were generated for the two strains isolated from humans in Europe in 1990, termed PAli and PAbe (fifth and sixth *in vitro* passages, respectively) (1, 2), and of strain B31 originating from an *Ixodes scapularis* tick in North America (3, 4). This provided the possibility to explore whether the similarities of these strains were confined to the conserved main chromosome or whether they extended to plasmids present in these strains. Strain B31 sequenced here [termed B31(NRZ)] originates from the same biological source as strain B31, published by Fraser et al. (4). The difference between the two strains is that the B31 was passed through a mouse before sequencing (5).

We sequenced the complete genomes of low-passage-number cultures of the strains via Illumina MiSeq and Pacific Biosciences system technologies. For Illumina sequencing, libraries were constructed using Nextera XT, TruSeq, and mate-pair libraries. Library construction, Pacific Biosciences single-molecule real-time (SMRT) sequencing, and contig assembly were performed at the Genome Sequencing Unit of the University of Oslo (6). For gap closure and construction of completed genomes, PacBio SMRT assemblies were used as a reference for read mapping of Illumina sequences using the CLC Genomics Workbench (Qiagen, Germany). The following settings were used for read mapping: mismatch cost = 2, cost of insertions and deletions = linear gap cost, length fraction = 0.5, similarity fraction = 0.8, autodetect paired distances = yes, and nonspecific match handling = map randomly. Variant calls were generated using the fixed ploidy variant detection option with ploidy = 1, required variant probability = 90%, minimum coverage = 10×, minimum count = 2, minimum frequency = 80%, base quality filter = yes, neighborhood radium = 5, minimum central quality = 20, and minimum neighborhood quality = 15. Low-coverage regions were filled from the reference sequence. Uncertain SNPs were examined manually and if required corrected.

Genomes were submitted to NCBI and annotation was conducted using the NCBI Prokaryotic Genome Annotation Pipeline. A total of 1,341,096 bases, 1,308,038 bases, and 1,301,535 bases for strains B31(NRZ), PAli, and PAbe, respectively, were assembled. The genomes of B31(NRZ), PAli, and PAbe contained 1,303, 1,232, and 1,259 coding genes, 40 RNA loci, and 73, 72, and 72 pseudogenes, respectively. B31(NRZ), PAli, and PAbe possessed a number of linear (6, 5, and 5, respectively) and circular (6, 6, and 5, respectively) plasmids. Unexpectedly, the data revealed that closely related strains (i.e., identical MLSTs) may reveal differences in their plasmids with unknown consequences for pathogenicity or ecology. The availability of these completed genomes is a major step toward a better understanding of the population structure of *B. burgdorferi sensu stricto* and human pathogenicity of strains.

### Accession number(s).

The number of replicons and accession numbers are presented in Table 1.

**TABLE 1:** NCBI accession numbers

Replicon name	Accession no. by strain		
	B31(NRZ)	PAli	PAbe
Main chromosome	CP019767	CP019844	CP019916
cp26	CP019755	CP019845	CP019917
cp32-1		CP019846	
cp32-5+1	CP019756		CP019918
cp32-2	CP019757		CP019919
cp32-3	CP019758	CP019847	CP019920
cp32-4	CP019759	CP019848	
cp32-5		CP019849	
cp32-9	CP019760	CP019850	
cp32-9+4			CP019921
lp17	CP019761	CP019851	CP019922
lp28-1	CP019762		CP019923
lp36	CP019763	CP019852	CP019924
lp38	CP019764	CP019853	
lp54	CP019765	CP019854	CP019925
lp56	CP019766	CP019855	CP019926

## References

[1] Jungnick S, Margos G, Rieger M, Dzaferovic E, Bent SJ, Overzier E, Silaghi C, Walder G, Wex F, Koloczek J, Sing A, Fingerle V 2015 *Borrelia burgdorferi sensu stricto* and *Borrelia afzelii*: population structure and differential pathogenicity. Int J Med Microbiol 305:673–681. doi:10.1016/j.ijmm.2015.08.017. 26341331

[2] Castillo-Ramírez S, Fingerle V, Jungnick S, Straubinger RK, Krebs S, Blum H, Meinel DM, Hofmann H, Guertler P, Sing A, Margos G 2016 Trans-Atlantic exchanges have shaped the population structure of the Lyme disease agent *Borrelia burgdorferi sensu stricto*. Sci Rep 6:22794. doi:10.1038/srep22794. 26955886 PMC4783777

[3] Burgdorfer W, Barbour AG, Hayes SF, Benach JL, Grunwaldt E, Davis JP 1982 Lyme disease-a tick-borne spirochetosis? Science 216:1317–1319. doi:10.1126/science.7043737. 7043737

[4] Fraser CM, Casjens S, Huang WM, Sutton GG, Clayton R, Lathigra R, White O, Ketchum KA, Dodson R, Hickey EK, Gwinn M, Dougherty B, Tomb JF, Fleischmann RD, Richardson D, Peterson J, Kerlavage AR, Quackenbush J, Salzberg S, Hanson M, van Vugt R, Palmer N, Adams MD, Gocayne J, Weidman J, Utterback T, Watthey L, McDonald L, Artiach P, Bowman C, Garland S, Fuji C, Cotton MD, Horst K, Roberts K, Hatch B, Smith HO, Venter JC 1997 Genomic sequence of a Lyme disease spirochaete, *Borrelia burgdorferi*. Nature 390:580–586. doi:10.1038/37551. 9403685

[5] Casjens S, van Vugt R, Tilly K, Rosa PA, Stevenson B 1997 Homology throughout the multiple 32-kilobase circular plasmids present in Lyme disease spirochetes. J Bacteriol 179:217–227. doi:10.1128/jb.179.1.217-227.1997. 8982001 PMC178682

[6] Margos G, Hepner S, Mang C, Marosevic D, Reynolds SE, Krebs S, Sing A, Derdakova M, Reiter MA, Fingerle V 2017 Lost in plasmids: next generation sequencing and the complex genome of the tick-borne pathogen Borrelia burgdorferi. BMC Genomics 18:422. doi:10.1186/s12864-017-3804-5. 28558786 PMC5450258

